# The somatostatin receptor 2 antagonist ^64^Cu-NODAGA-JR11 outperforms ^64^Cu-DOTA-TATE in a mouse xenograft model

**DOI:** 10.1371/journal.pone.0195802

**Published:** 2018-04-18

**Authors:** Svetlana N. Rylova, Christian Stoykow, Luigi Del Pozzo, Keelara Abiraj, Maria Luisa Tamma, Yvonne Kiefer, Melpomeni Fani, Helmut R. Maecke

**Affiliations:** 1 German Cancer Consortium (DKTK), Heidelberg, Germany; 2 Department of Nuclear Medicine, Medical Center–University of Freiburg, Faculty of Medicine, University of Freiburg, Freiburg, Germany; 3 German Cancer Research Center (DKFZ), Heidelberg, Germany; 4 Division of Radiological Chemistry, University Hospital Basel, Basel, Switzerland; Monash University, AUSTRALIA

## Abstract

Copper-64 is an attractive radionuclide for PET imaging and is frequently used in clinical applications. The aim of this study was to perform a side-by-side comparison of the *in vitro* and *in vivo* performance of ^64^Cu-NODAGA-JR11 (NODAGA = 1,4,7-triazacyclononane,1-glutaric acid,4,7-acetic acid, JR11 = p-Cl-Phe-cyclo(D-Cys-Aph(Hor)-D-Aph(cbm)-Lys-Thr-Cys)D-Tyr-NH_2_), a somatostatin receptor 2 antagonist, with the clinically used sst2 agonist ^64^Cu-DOTA-TATE ((TATE = D-Phe-cyclo(Cys-Tyr-D-Trp-Lys-Thr-Cys)Thr). *In vitro* studies demonstrated K_d_ values of 5.7±0.95 nM (Bmax = 4.1±0.18 nM) for the antagonist ^64/nat^Cu-NODAGA-JR11 and 20.1±4.4. nM (Bmax = 0.48±0.18 nM) for the agonist ^64/nat^Cu-DOTA-TATE. Cell uptake studies showed the expected differences between agonists and antagonists. Whereas ^64^Cu-DOTA-TATE (the agonist) showed very effective internalization in the cell culture assay (with 50% internalized at 4 hours post-peptide addition under the given experimental conditions), ^64^Cu-NODAGA-JR11 (the antagonist) showed little internalization but strong receptor-mediated uptake at the cell membrane. Biodistribution studies of ^64^Cu-NODAGA-JR11 showed rapid blood clearance and tumor uptake with increasing tumor-to-relevant organ ratios within the first 4 hours and in some cases, 24 hours, respectively. The tumor washout was slow or non-existent in the first 4 hours, whereas the kidney washout was very efficient, leading to high and increasing tumor-to-kidney ratios over time. Specificity of tumor uptake was proven by co-injection of high excess of non-radiolabeled peptide, which led to >80% tumor blocking. ^64^Cu-DOTA-TATE showed less favorable pharmacokinetics, with the exception of lower kidney uptake. Blood clearance was distinctly slower and persistent higher blood values were found at 24 hours. Uptake in the liver and lung was relatively high and also persistent. The tumor uptake was specific and similar to that of ^64^Cu-NODAGA-JR11 at 1 h, but release from the tumor was very fast, particularly between 4 and 24 hours. Tumor-to-normal organ ratios were distinctly lower after 1 hour. This is indicative of insufficient *in vivo* stability. PET studies of ^64^Cu-NODAGA-JR11 reflected the biodistribution data with nicely delineated tumor and low background. ^64^Cu-NODAGA-JR11 shows promising pharmacokinetic properties for further translation into the clinic.

## Introduction

Radiolabeled somatostatin receptor agonists readily internalize into tumor cells *in vitro* [1] and *in vivo* [2], allowing active accumulation of radioactivity in tumor cells. Neutral antagonists do not internalize and were not originally considered as targeting agents for tumor localization and targeted radionuclide therapy. However, antagonists often recognize more binding sites because they can target a variety of active and inactive conformations of G-protein-coupled receptors (GPCRs) [3,4], indicating that they may be promising targeting agents for imaging and targeted radionuclide therapy. Indeed, Ginj et al have found that radiolabeled, chelator-coupled sst2- and sst3-selective antagonists do not trigger receptor internalization but still show excellent *in vivo* tumor uptake and retention [5]. These features have been further confirmed with different somatostatin receptor-targeting peptide probes employing different chelators and radiometals [6]. Importantly, the ^111^In- and ^177^Lu-labeled somatostatin-based peptidic antagonists have been successfully translated into the clinic for imaging neuroendocrine tumors [7,8].

In addition, the preclinical studies have recently been extended to antagonistic peptides labeled with ^64^Cu [9]. In recent years, Copper-64 has gained popularity in nuclear medicine primarily because of its longer half-life (t_1/2_ = 12.7 hours), which enables PET imaging at later time points with higher tumor-to-normal organ contrasts [10]. In addition, ^64^Cu has the potential for theranostic applications when paired with ^67^Cu (t_1/2_ = 61.9 hours; *β*^*-*^, E_max_ = 0.141 MeV [100%]), suitable for targeted radionuclide therapy. Furthermore, ^64^Cu can be manufactured in a central facility carrier-free in high amounts on a medical cyclotron via the ^64^Ni(p,n)^64^Cu reaction and distributed to distant hospitals [11].

Since efficient labeling—including fast formation kinetics (high labeling yields at low concentrations) and high kinetic and thermodynamic stability—is of paramount importance for a clinical PET tracer, efforts were made to develop suitable bifunctional chelators and conjugation methods for biomolecules [12–16]. The literature on radiocopper-based radiopharmaceuticals in general was summarized by Hao et al [17] and Shokeen et al [10,18], while the literature on radiocopper-labeled somatostatin analogues was recently summarized by Brasun et al [9].

We have shown that the ^64^Cu-labeled somatostatin receptor 2 antagonist LM3(p-Cl-Phe-cyclo(D-Cys-Tyr-D-Aph(Cbm)-Lys-Thr-Cys)D-Tyr-NH_2_) exhibits excellent pharmacokinetics, including high tumor uptake and high tumor-to-background ratios when conjugated to the established Cu(II) chelators NODAGA and CB-TE2A [6].

Recently, we have studied several sst2 antagonists and selected DOTA-JR11 (JR11: p-Cl-Phe-cyclo(D-Cys-Aph(Hor)-D-Aph(Cbm)-Lys-Thr-Cys)D-Tyr-NH_2_) as the favored peptide for clinical translation, as it showed high receptor affinity when labeled with ^90^Y and ^177^Lu for targeted radionuclide therapy. In addition, the log D value of ^177^Lu-DOTA-JR11 is approximately -2.5, very similar to radiometal-labeled DOTATOC ([DOTA,Tyr^3^]octreotide) and DOTATATE ([DOTA,Tyr^3^,Thr^8^]octreotide, indicating high hydrophilicity [19]. However, we found that the affinity of DOTA-JR11 labeled with ^68^Ga or ^64^Cu was lower than the affinity for other tested JR-11 radioconjugates (29 ± 2.7 nM and 16 ± 1.2 nM, respectively, versus 0.47–3.8 nM for other conjugates). Furthermore, using the NODAGA chelator instead of DOTA dramatically improved the affinity of ^68^Ga-labeled JR-11 conjugate [19]. Accordingly, we hypothesized that the affinity of the ^64^Cu-labeled JR11 candidate could also benefit from using the NODAGA chelator. Based on our experience, NODAGA is a very good chelator for ^64^Cu. It confers high thermodynamic, kinetic, redox stability and very favorable pharmacokinetics for a number of small peptides [6]. The aim of the current study was to evaluate *in vitro* and *in vivo* the ^64^Cu-labeled NODAGA-JR11 sst2 targeting probe and perform a side-by-side comparison with ^64^Cu-DOTA-TATE ([^64^Cu-DOTA, Tyr^3^, Thr^8^]octreotide, which has been tested very successfully in the clinic [20,21]. ^64^Cu-DOTA-TATE was recently shown to be far superior to SRS (somatostatin receptor scintigraphy) with ^111^In-octreoscan [21]. In addition, a head-to-head comparison of ^64^Cu-DOTA-TATE and ^68^Ga-DOTA-TOC PET/CT showed significantly more lesions in a cohort of 59 patients with the ^64^Cu-labeled radiopeptide [20].

## Materials and methods

### Materials

All starting reagents listed were obtained from commercial sources and used without further purification. Amino acids and the Rink amide methyl-benzhydrylamine (MBHA) resin were purchased from NovaBiochem (Darmstadt, Germany) and Bachem AG, (Duebendorf, Switzerland). Copper-64 chloride (^64^CuCl_2_) was available from University Hospital Tübingen, Germany. (R)-NODAGA(t-Bu)_3_ was purchased from CheMatec (Dijon, France).

### Synthesis of peptides, coupling to chelators, and (radio)metal complexation

The unnatural amino acids D-Aph(Cbm) (d-4-amino-Phe-carbamoyl), a D-Trp mimetic and Aph(Hor) (4-amino-L-hydroorotyl-phenylalanine) mimicking Tyr, were synthesized as described earlier [22]. The peptide analogs and the corresponding DOTA- and NODAGA-conjugates were synthesized following standard solid-phase peptide synthesis on a methyl-benzhydrylamine resin (MBHA) as previously described [17]. DOTA-TATE was synthesized as published earlier [23]. The final products of NODAGA-JR11 and DOTA-TATE were purified by semi-preparative HPLC and characterized by electrospray ionization mass spectrometry (ESI-MS) and reverse-phase high-performance liquid chromatography (RP-HPLC).

Copper-64 labeling of (R)-NODAGA-JR11 and DOTA-TATE was done in ammonium acetate buffer (0.1 M, pH 8.0) at 95°C for 10 minutes. For saturation binding experiments, one equivalent of ^nat^CuCl_2_ was added after labeling and the reaction mixture was incubated for another 10 minutes at 95°C. Quality control of radiolabeled peptides was performed by reversed-phase high-performance liquid chromatography (RP-HPLC) as described previously [19].

### Receptor binding and internalization studies

All cell experiments were performed in a human embryonic kidney HEK-293 cell line stably expressing human sst2 receptors (HEK-hsst2) (a gift from Prof. Schulz, University of Jena) plated in 6-well plates in triplicates (10^6^ cells/well). The saturation binding experiments were performed with radioligand (^64/nat^Cu-NODAGA-JR11 and ^64/nat^Cu-DOTA-TATE) concentrations ranging from 0.5–75 nM and 0.5–90 nM, respectively, for 2 hours at +4ºC as described previously [24]. The K_d_ and Bmax values were calculated using GraphPad.

The internalization rate of the agonist ^64^Cu-DOTA-TATE was studied after addition of 2.5 pmol of ^64^Cu-DOTA-TATE to the medium of the cells followed by incubation (in triplicates) for 0.5, 1, 2, and 4 h at 37°C, 5% CO_2_. Non-specific, surface-bound, and internalized radiopeptides were determined in the presence of 10 μM TATE. The final volume was 1.5 mL/well. At the indicated time points, the cellular uptake was stopped by removal of the medium and washing the cells with ice-cold PBS (pH 7.4). Cells were then treated three times for 5 min with glycine buffer (0.05 mol/L glycine solution, pH 2.8) to distinguish between cell surface-bound (acid-releasable) and internalized (acid-resistant) radioligand. Finally, cells were detached from the plates by incubation with 1 mol/L NaOH for 10 min at 37°C. The radioactivity of all fractions was measured in a γ-counter.

### Cellular retention studies: Dissociation/Externalization

The dissociation rate of the antagonist ^64^Cu-NODAGA-JR11 was studied in HEK-hsst2 cells after incubation with 2.5 pmol of ^64^Cu-NODAGA-JR11/mL/well (specific activity 40 MBq/nmol) for 2 hours on ice. The low temperature prevents internalization while the long incubation time ensures equilibrium. After 2 hours, the unbound radioligand was rinsed off with cold medium and the cells were treated with 0.9 mL of pre-warmed medium (37°C) along with 0.1 mL of NODAGA-JR-11 (50 pmol) or PBS. The 6-well plates were then immediately transferred to 37ºC. After 10, 20, 30, and 60 minutes at 37ºC, the medium, which contained dissociated radioligand, was removed for quantification. The surface-bound and internalized fractions were obtained as described above and quantified in the γ-counter.

The externalization rate of the agonist ^64^Cu-DOTA-TATE was studied after adding 2.5 pmol/mL/well of ^64^Cu-DOTA-TATE (specific activity 7 MBq/nmol) to HEK-hsst2 cells and incubation for 2 hours at 37°C to allow internalization; cells were then washed twice with PBS and the receptor-bound ligands were removed by washing with glycine buffer (pH 2.8). Next, cells were incubated with fresh medium at 37°C for indicated time periods and the radioactivity retained in the cell after 10, 20, 30, 60, and 120 minutes was measured.

### Biodistribution in HEK-hsst2-bearing animals

All animal experiments were conducted in accordance with the German Animal Welfare Act (TierSchG). The protocol was approved by the Animal Welfare Ethics committees of the University of Freiburg (Permit Number: G-16/02). Female Balb/c nude mice (18–20 g, 6–8 weeks old) were obtained from Janvier Labs (Saint-Berthevin Cedex, France) and were housed and handled in accordance with good animal practice as defined by FELASA and the national animal welfare body GVSOLAS. Xenografts were established on the right shoulder by s.c. injection of 10 million Hek-hsst2 cells freshly suspended in 100 μL sterile PBS. The tumors were allowed to grow for 14–18 days (tumor weight: 250–350 mg).

For biodistribution, Hek-hsst2 tumor-bearing mice were administered 10 pmol/100 μL/0.4 MBq of ^64^Cu-NODAGA-JR11 or 10 pmol/100ul/0.6 MBq of ^64^Cu-DOTA-TATE via the tail vein and were euthanized at 1 hour, 4 hours, and 24 hours post-injection (p.i.). For mass dependence experiments, mice were injected with increasing masses of ^64^Cu-NODAGA-JR11 (200 pmol, 1,000 pmol, and 2,000 pmol) and biodistribution was studied at 1 hour p.i. Non-specific uptake of ^64^Cu-NODAGA-JR11 and ^64^Cu-DOTA-TATE radiopeptides was determined by co-injection with 10 nmol of NODAGA-JR11 or 20 nmol of TATE, respectively. Organs of interest and blood were collected, rinsed of excess blood, blotted dry, weighted, and counted in a γ-counter. The results were expressed as a percentage of injected activity per gram tissue (%IA/g) and represent the mean±SD of n = 3–4. The total counts injected per mouse were determined by extrapolation from counts of a known aliquot of the injected solution.

### Small-animal PET studies

PET scans were performed using a dedicated small-animal PET scanner (Focus 120 microPET scanner; Concorde Microsystems, Inc.). ^64^Cu-NODAGA-JR11 (8 MBq/200 pmol) was administered to two mice with HEK-hsst2 tumor xenografts, as described above. Animals were anesthetized with 1.8% isoflurane and static scans were acquired at 1 hour, 4 hours, and 24 hours p.i. for 20 to 30 minutes. Additionally, 2 mice injected with different peptide masses of ^64^Cu-NODAGA-JR11 (1,000 pmol, n = 2; 2,000 pmol, n = 2) were imaged at 1 hour p.i. PET images were reconstructed with filtered back projection. No correction was applied for attenuation. Maximum intensity projection (MIP) PET images were generated using Rover software. The color scale was set from 0 to 10% to allow for qualitative comparison among the images.

## Results

### Synthesis and radiochemistry

**Fig 1:**
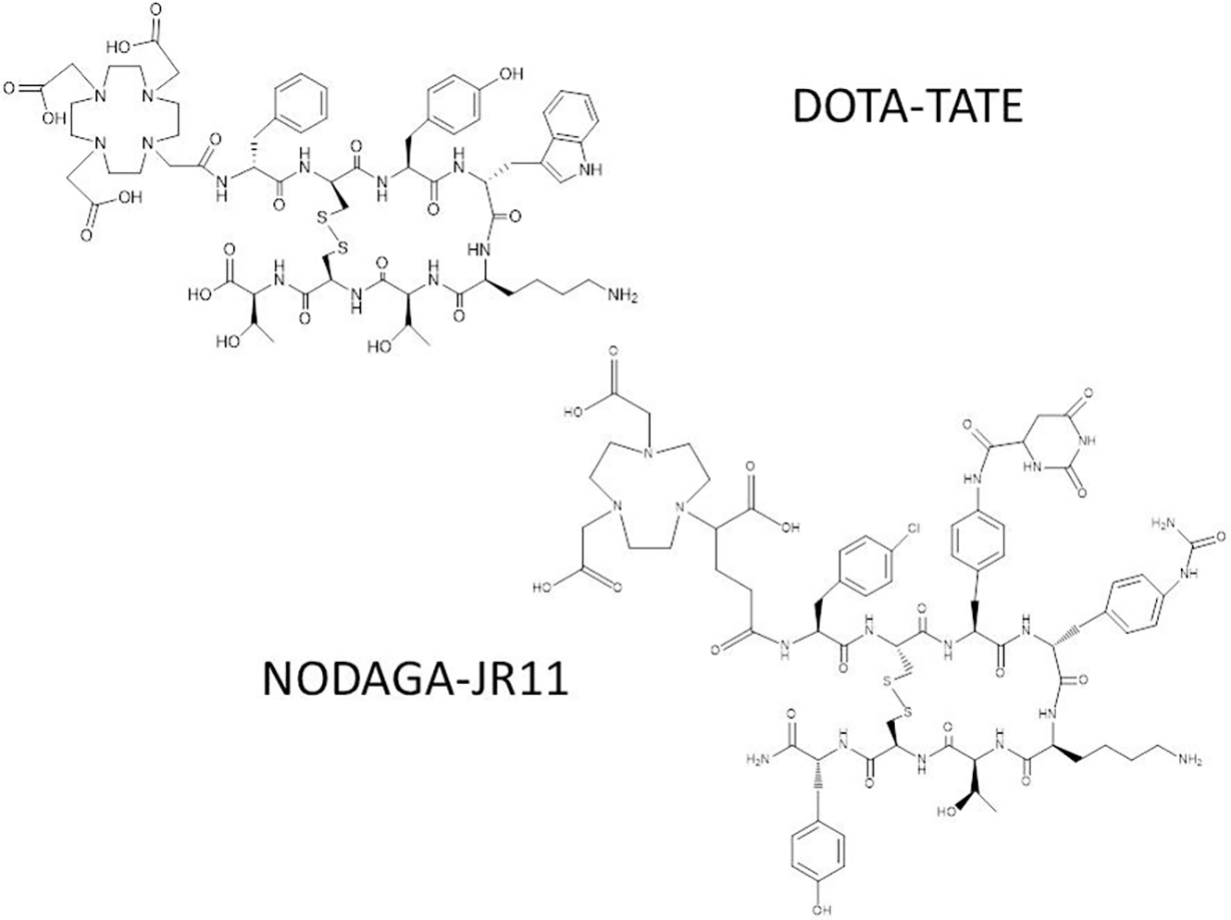
Chemical structures of NODAGA-JR11 and DOTA-TATE.

NODAGA-JR11 and DOTA-TATE (Fig 1) were generated via solid-phase peptide synthesis using Fmoc strategy as published elsewhere. The final yield was about 40% based on the first Fmoc removal (6). The purity as determined by RP-HPLC was >97%.

^64^Cu-NODAGA-JR11 was synthesized with 99% labeling yield and a specific activity of 40 MBq/nmol (S1A Fig). ^64^Cu-DOTA-TATE was synthesized with a labeling yield of 97% and specific activity of 7 MBq/nmol (S1B Fig).

### Binding affinity studies

**Fig 2:**
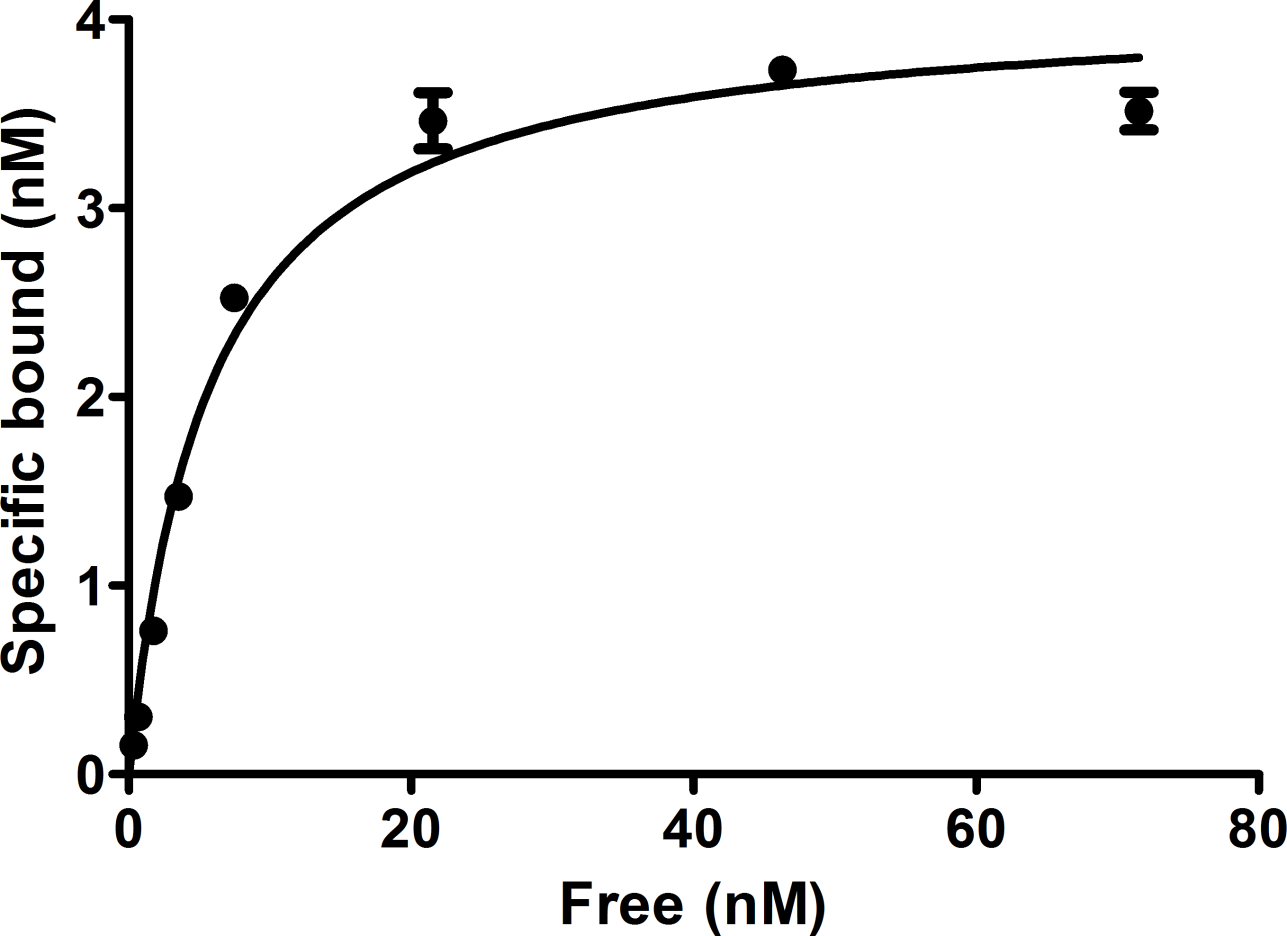
Saturation binding of ^64^Cu-NODAGA-JR11 to HEK-hsst2 cells. The cells were incubated with increasing concentrations of ^64/nat^Cu-NODAGA-JR11 (range 0.5–75 nM) for 2 hours at +4ºC.

**Fig 3:**
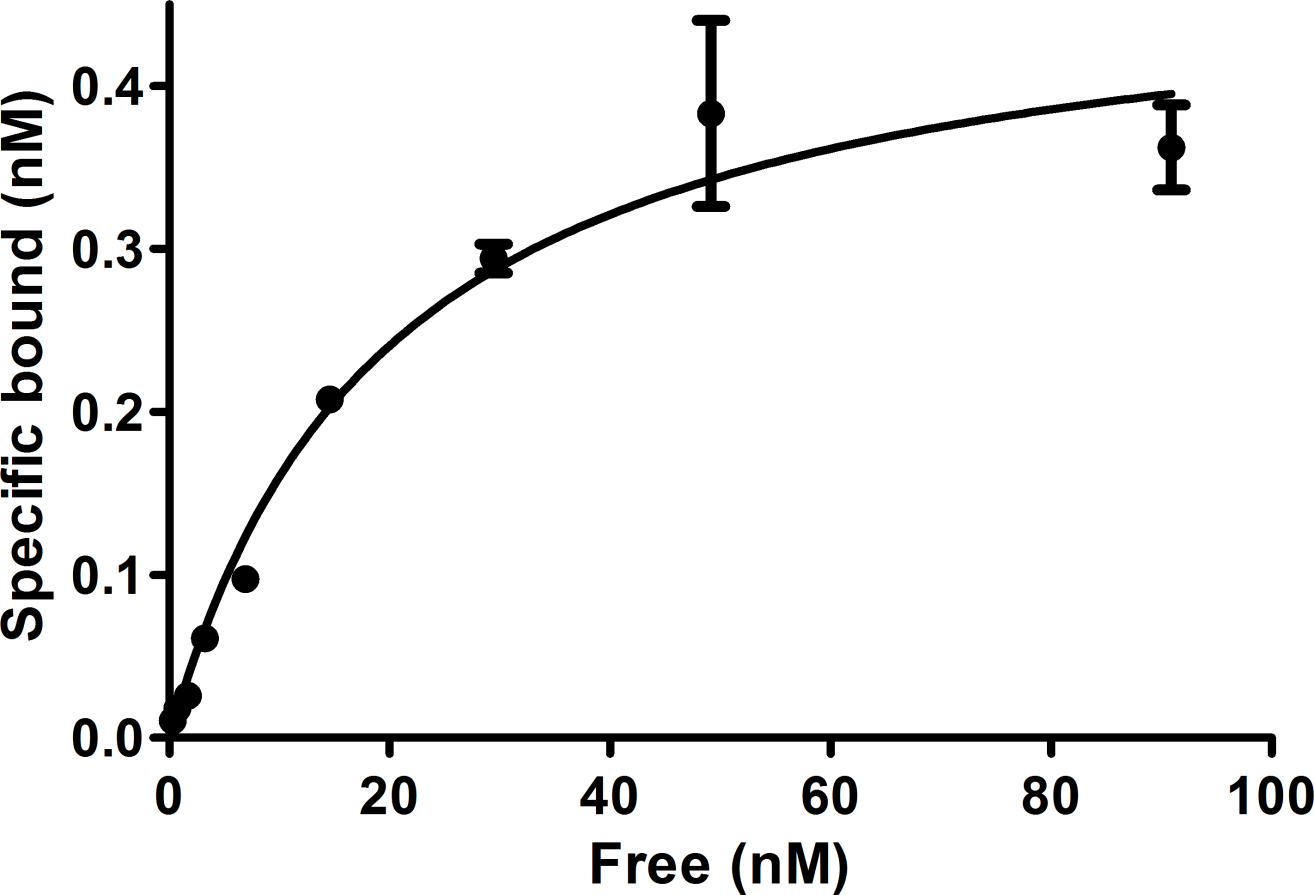
Saturation binding of ^64/nat^Cu-DOTA-TATE onto HEK-hsst2 cells. The cells were incubated with increasing concentrations of ^64^Cu-DOTA-TATE (range 0.5–90 nM) for 2 hours at +4ºC.

The dissociation constants were determined on HEK-hsst2 cells using ^64/nat^Cu-NODAGA-JR11 and ^64/nat^Cu-DOTA-TATE as receptor ligands at 4°C via saturation binding (Figs 2 and 3). The K_d_ value for ^64/nat^Cu-NODAGA-JR11 was calculated to be 5.7 ± 0.95 nM and Bmax was 4.1 ± 0.18 nM. The K_d_ and Bmax values for ^64/nat^Cu-DOTA-TATE were 20.1 ± 4.4 nM and 0.48 ± 0.04 nM, respectively.

### *In vitro* cellular uptake and retention

**Fig 4:**
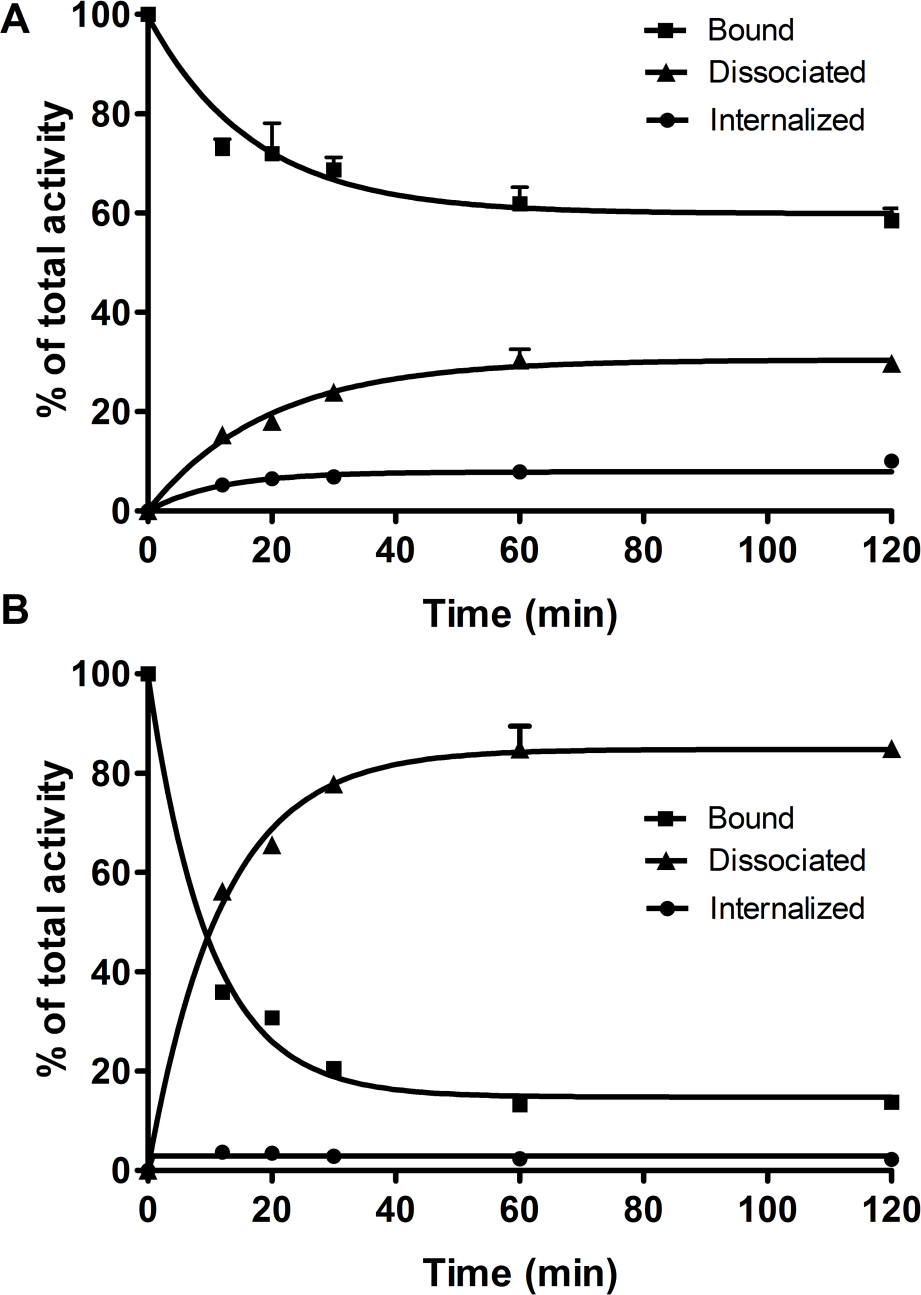
Internalization, binding, and dissociation of ^64^Cu-NODAGA-JR11. HEK-hsst2 cells were incubated with ^64^Cu-NODAGA-JR11 at +4°C for 120 minutes and the temperature was shifted to 37°C by adding warm media (A) or media containing 50 pmol of NODAGA-JR11 (B). The amount of cell-bound, internalized, and dissociated activity was determined between 0 to 120 minutes after the temperature shift.

An important parameter with regard to long retention of non-internalizing antagonistic radiopeptides in particular is the rate of dissociation from the receptor. The experiment with ^64^Cu-NODAGA-JR11 was performed as a temperature shift experiment. The radioligand was allowed to bind for 2 hours at 4°C, followed by a quick shift to 37°C. In culture medium, ^64^Cu-NODAGA-JR11 dissociated from the receptor relatively quickly but reached a steady state after only about 1 hour at ca 60% bound radiopeptide (Fig 3). The remaining radioligand was either in the medium (dissociated, ca 30%) or internalized (ca 10%) (Fig 4). The relative amounts were dependent on the presence of unlabeled peptide. With 50 pmol of NODAGA-JR11 in the medium, the plateau was reached at about the same time but at a much lower level, at about 20% ^64^Cu-NODAGA-JR11 still remaining receptor bound. Very little ^64^Cu-NODAGA-JR11 was internalized (<5%).

**Fig 5:**
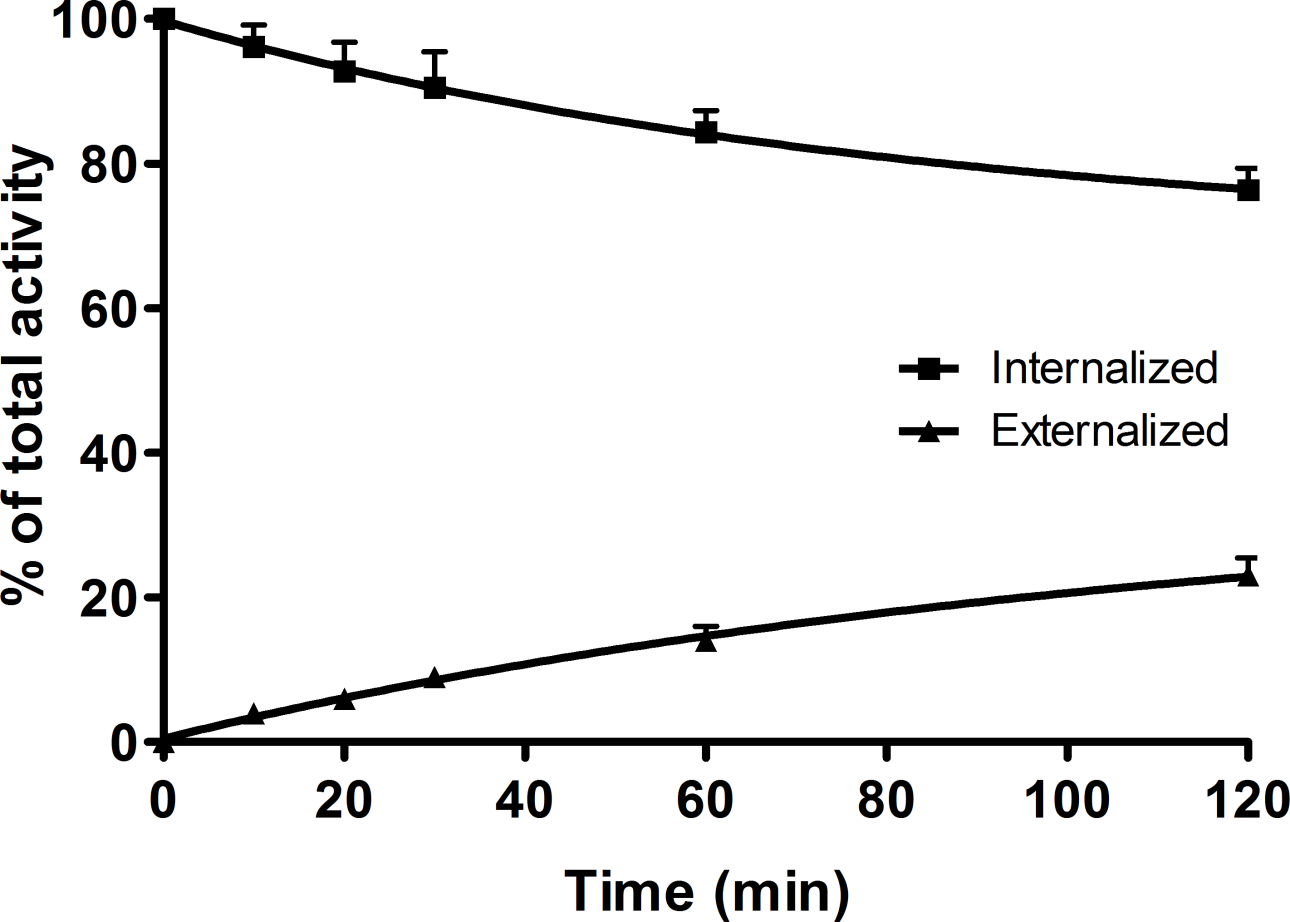
Externalization kinetics of ^64^Cu-DOTA-TATE in HEK-hsst2 cells. HEK-hsst2 cells were incubated with ^64^Cu-DOTA-TATE for 2 hours at +37°C. The receptor-bound ligands were removed by washing with glycine buffer, pH 2.8. Cells were then incubated by adding fresh medium and the amount of externalized and internalized (retained) radioactivity in the cells was measured after 10, 20, 30, 60, and 120 minutes.

The agonist ^64^Cu-DOTA-TATE internalized into the HEK-hsst2 cells with a steadily increasing amount of internalized peptide from 21.26 ± 1.23% at 30 minutes to 50.95 ± 4.30% at 4 hours (S2 Fig). No plateau was reached under the given conditions. The cellular retention of ^64t^Cu-DOTA-TATE was studied in an externalization experiment. The amount of retained/internalized peptide reached the plateau of 80% after 2 hours of incubation at 37°C. The remaining 20% of radioactivity was externalized into the medium (Fig 5).

### *In vivo* biodistribution results

The biodistribution of ^64^Cu-NODAGA-JR11 and ^64^Cu-DOTA-TATE was studied at 1, 4, and 24 hours p.i. using 10 pmol of total peptide mass. Tables 1 and 2 summarize the pharmacokinetics of the two radiopeptides. ^64^Cu-NODAGA-JR11 showed a fast blood clearance with only 0.1 ± 0.0%IA/g remaining in the blood at 4 hours p.i. It accumulated in the tumor, kidneys, and sst2-positive organs, such as stomach, adrenals, and pancreas (Table 1). The tumor uptake was 20.6 ± 3.7%IA/g at 1 hour p.i. and remained essentially the same between 1 and 4 hours p.i. (19.0 ± 3.1%IA/g). However, significant washout was found within 24 hours (7.7 ± 2.5%IA/g). A high accumulation of radioactivity was also found in the kidneys (10.3 ± 0.7%IA/g at 1 hour p.i.), which decreased to 2.2 ± 0.6%IA/g within 24 hours (Table 1). The tumor:normal organ ratios were increasing from 1 to 4 hours by a factor of 1.4 up to 13, depending on the background tissue. The tumor:kidney uptake ratio increased further at 24 hours p.i.

**Table 1:** Biodistribution of 10 pmol of ^64^Cu-NODAGA-JR11 in mice bearing HEK-hsst2 xenografts.

Organ	%IA/g^#^			
	1 h	1 h blocking^a^	4 h	24 h
**Blood**	0.6±0.2	0.3±0.0	0.1±0.0	0.1±0.0
**Heart**	0.5±0.1	0.3±0.1	0.3±0.1	0.2±0.1
**Lung**	3.0±1.0	0.9±0.2	0.8±0.2	0.5±0.1
**Liver**	1.7±0.2	1.3±0.0	0.7±0.3	0.3±0.1
**Spleen**	0.9±0.2	0.5±0.0	1.2±0.2	0.9±0.1
**Pancreas**	5.6±0.6	0.2±0.0	0.4±0.1	0.2±0.0
**Stomach**	4.9±1.6	0.7±0.0	0.9±0.2	0.4±0.0
**Intestine**	0.9±0.4	0.7±0.1	0.7±0.1	0.4±0.0
**Kidney**	10.3±0.7	6.4±1.3	6.9±0.9	2.2±0.6
**Adrenal**	4.8±0.8	3.7±1.0	3.5±0.9	1.5±0.6
**Muscle**	0.4±0.0	0.4±0.2	0.1±0.1	0.04±0.0
**Bone**	0.7±0.2	0.5±0.0	0.8±0.2	0.4±0.2
**Tumor**	20.6±3.7	3.9±0.0	19.0±3.1	7.7±2.5
**Tumor:Blood**	29.4		190.0	77.0
**Tumor:Pancreas**	3.7		47.5	38.5
**Tumor:Stomach**	4.2		21.1	19.3
**Tumor:Intestine**	22.9		27.1	19.3
**Tumor:Liver**	12.1		27.1	25.7
**Tumor:Kidney**	2.0		2.8	3.5
**Tumor:Muscle**	51.5		190.0	192.5

^#^Values are mean±standard deviation (*n* = 3–4).

^a^For blocking, 10 nmol of unlabeled peptide was used.

**Table 2:** Biodistribution of 10 pmol of ^64^Cu-DOTA-TATE in mice bearing HEK-hsst2 xenografts.

Organ	%IA/g^#^			
	1 h	4 h	4 h blocking^a^	24 h
**Blood**	0.7±0.2	0.6±0.1	0.5±0.04	0.5±0.05
**Heart**	1.1±0.1	1.2±0.3	1.3±0.4	1.1±0.2
**Liver**	6.1±1.2	5.2±1.6	6.2±1.2	4.6±0.6
**Spleen**	1.7±0.6	1.1±0.4	1.01±0.5	1.2±0.1
**Lung**	9.8±2.6	5.4±1.4	3.6±1.5	4.2±0.6
**Kidney**	4.8±0.5	3.0±0.6	4.1±1.5	2.7±0.3
**Stomach**	16.1±1.7	10.6±1.6	3.5±1.5	3.1±0.3
**Intestine**	4.9±0.2	3.6±0.4	3.3±0.9	2.0±0.3
**Adrenal**	4.7±1.5	3.3±1.1	2.3±0.9	0.7±0.3
**Pancreas**	24.6±2.4	4.4±1.0	1.2±0.4	0.95±0.1
**Muscle**	0.3±0.1	0.2±0.03	0.2±0.05	0.3±0.1
**Bone**	1.6±0.5	1.4±0.2	0.8±0.2	0.8±0.1
**Tumor**	20.3±2.5	14.9±1.0	2.6±0.9	2.8±0.23
**Tumor:Blood**	29.0	24.8		5.6
**Tumor:Pancreas**	0.8	3.4		2.9
**Tumor:Stomach**	1.3	1.4		0.9
**Tumor:Intestine**	4.1	4.1		1.4
**Tumor:Liver**	3.3	2.9		0.6
**Tumor:Kidney**	4.2	5.0		1.0
**Tumor:Muscle**	67.7	74.5		9.3

^#^Values are mean±standard deviation (*n* = 3–4).

^**a**^co-injection with 20 nmol of TATE

^64^Cu-DOTA-TATE showed slower clearance from the blood with 0.7 ± 0.2%IA/g at 1 hour p.i and 0.5 ± 0.05%IA/g still remaining after 24 hours p.i (Table 2). The tumor uptake of ^64^Cu-DOTA-TATE (20.3 ± 2.5%IA/g) was the same as that of ^64^Cu-NODAGA-JR11 (20.6 ± 3.7%IA/g) at 1 hour p.i., but decreased faster afterwards with only 2.8 ± 0.3%IA/g left at 24 hours p.i (Table 2). ^64^Cu-DOTA-TATE also showed relatively high accumulation and longer retention of activity in the normal organs, compared to ^64^Cu-NODAGA-JR11, including liver (6.1 ± 1.2 versus 1.7 ± 0.2%IA/g), pancreas (24.6 ± 2.4 versus 5.6 ± 0.6), stomach (16.1 ± 1.7 versus 4.9 ± 1.6%IA/g), and intestine (4.9 ± 0.2 versus 0.9 ± 0.4%IA/g) at 1 hour p.i. The kidney uptake was lower for ^64^Cu-DOTA-TATE (4.8 ± 0.5%IA/g at 1 hour p.i.) compared to ^64^Cu-NODAGA-JR11 (10.3 ± 0.7%IA/g at 1 hour p.i.). Contrary to what was seen for ^64^Cu-NODAGA-JR11, the tumor:normal organ ratios of ^64^Cu-DOTA-TATE did not increase from 1 to 4 hours p.i., but either remained at the same level or decreased. The only exception was the tumor:pancreas ratio, which improved by a factor of 4.3. At 24 hours p.i., all ratios decreased (Tables 1 and 2).

Between the two radiopeptides, the tumor:background ratios were already distinctly higher for ^64^Cu-NODAGA-JR11 compared to ^64^Cu-DOTA-TATE at 1 hour p.i., with the exception of the tumor:kidney ratio, which was favorable for ^64^Cu-DOTA-TATE (4.2 vs. 2.0). The difference in all tumor:normal organ ratios reached orders of magnitude within 4 hours p.i. in favor of ^64^Cu-NODAGA-JR11, while the difference in the tumor:kidney ratio was slightly reduced (5.0 vs. 2.8).

Blocking experiments confirmed the receptor-mediated uptake of ^64^Cu-NODAGA-JR11 and ^64^Cu-DOTA-TATE in the tumor and sst2-positive organs (Tables 1 and 2). Co-injection of 10 nmol corresponding unlabeled peptide led to 81% blocking of the tumor uptake for ^64^Cu-NODAGA-JR11, and 83% blocking for ^64^Cu-DOTA-TATE.

In order to elucidate if mass dependence has an influence on biodistribution as it was found for ^177^Lu-DOTA-JR11 [8], the biodistribution of ^64^Cu-NODAGA-JR11 was also studied with different masses (10 pmol, 200 pmol, 1000 pmol, and 2000 pmol) at 1 hour p.i. Among the studied peptide masses, 200 pmol of ^64^Cu-NODAGA-JR11 demonstrated the highest ratios of tumor to pancreas, stomach, intestine, and muscle at 1 hour p.i., while the tumor uptake remained almost the same (16.4 ± 0.7%IA/g) as with 10 pmol of the peptide. Further increase of the mass to 1000 and 2000 pmol also yielded good tumor-to-normal organ ratios, but there was a substantially lower uptake (50%) of activity in the tumor. The tumor-to-kidney ratio did not improve with any of the tested peptide masses (Table 3).

**Table 3:** Mass dependence of ^64^Cu-NODAGA-JR11 biodistribution in mice bearing HEK-hsst2 xenografts 1 hour p.i.

Organ	%IA/g ^#^			
	10 pmol	200 pmol	1000 pmol	2000 pmol
**Blood**	0.7±0.3	0.5±0.1	0.4±0.1	0.3±0.1
**Heart**	0.6±0.1	0.3±01	0.3±0.1	0.2±0.1
**Lung**	3.0±1.0	0.4±0.2	0.3±0.1	0.3±0.0
**Liver**	1.7±0.2	1.6±0.3	1.0±0.2	0.8±0.1
**Spleen**	0.9±0.2	0.7±0.1	0.5±0.1	0.6±0.1
**Pancreas**	5.6±0.6	1.7±0.3	0.8±0.2	0.6±0.1
**Stomach**	4.9±1.6	1.7±0.7	0.9±0.2	0.8±0.1
**Intestine**	0.9±0.4	0.5±0.1	0.3±0.1	0.4±0.1
**Kidney**	10.3±0.7	10.6±1.6	7.9±0.4	8.1±0.7
**Adrenal**	4.8±0.8	2.1±1.2	1.4±0.6	1.9±0.7
**Muscle**	0.4±0.0	0.2±0.0	0.3±0.2	0.2±0.0
**Bone**	1.3±0.7	0.5±0.3	0.8±0.4	0.2±0.0
**Tumor**	20.6±4.2	16.4±0.7	9.7±2.4	9.2±0.4
**Tumor:Blood**	29.4	32.8	24.2	30.7
**Tumor:Pancreas**	3.7	9.6	12.1	15.3
**Tumor:Stomach**	4.2	9.6	10.8	11.5
**Tumor:Intestine**	22.8	32.8	32.3	23.0
**Tumor:Liver**	12.1	10.3	9.7	11.5
**Tumor:Kidney**	2.0	1.5	1.2	3.5
**Tumor:Muscle**	51.5	82	32.3	192.5

^#^Values are mean±standard deviation (*n* = 3–4).

### PET imaging studies

**Fig 6:**
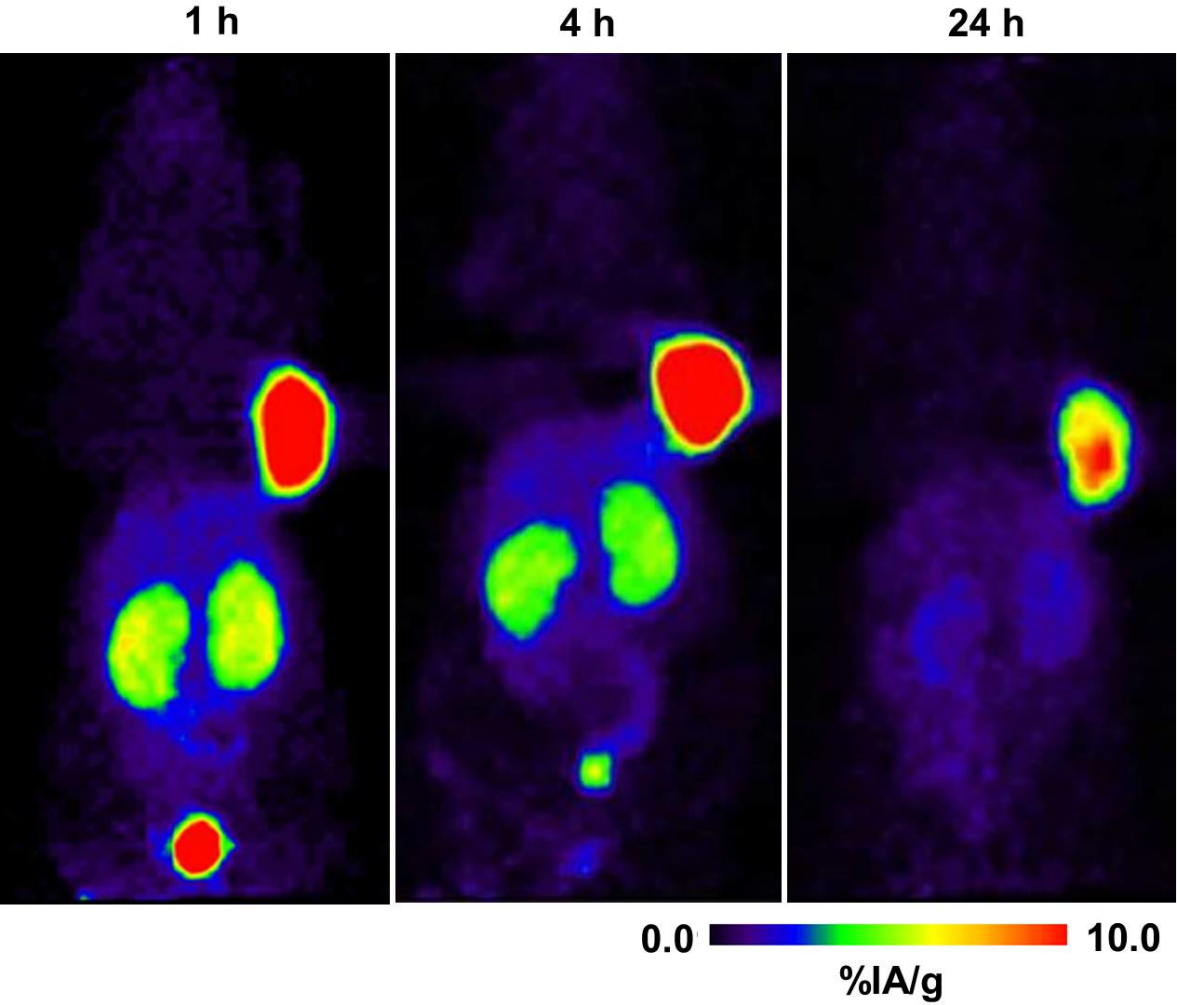
PET images of ^64^Cu-NODAGA-JR11 distribution in HEK-hsst2 tumor-bearing mice. Imaging data are presented as maximum intensity projections (MIP) at 1, 4, and 24 hours post-injection of 200 pmol of ^64^Cu-NODAGA-JR11.

**Fig 7:**
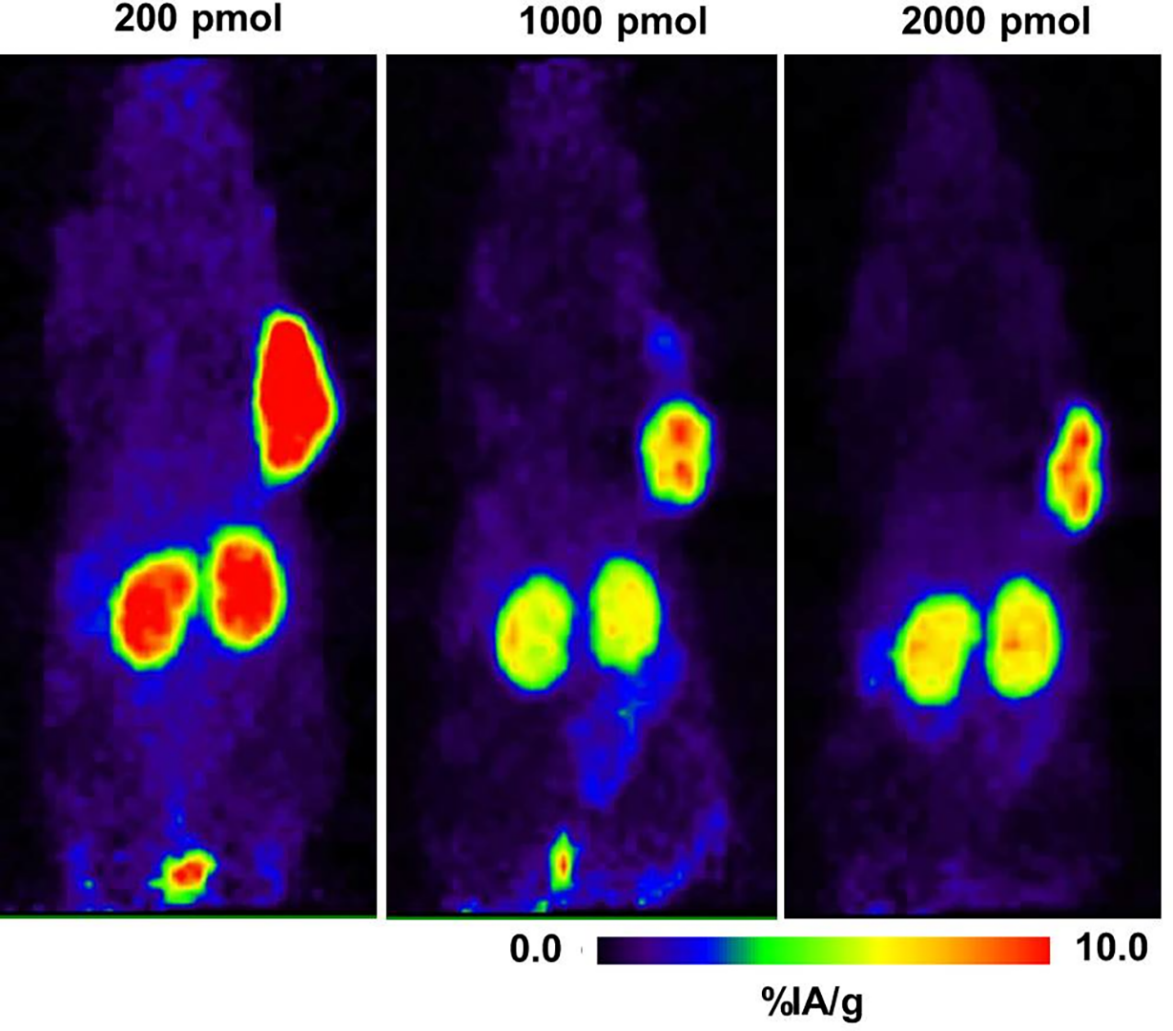
Mass dependence of the ^64^Cu-NODAGA-JR11 biodistribution in mice bearing HEK-hsst2 xenografts. Imaging data are presented as MIP at 1 hour post-injection of 200, 1000, and 2000 pmol of ^64^Cu-NODAGA-JR11.

PET imaging studies were performed with ^64^Cu-NODAGA-JR11, which has demonstrated the most favorable biodistribution profile. MIP PET images show that ^64^Cu-NODAGA-JR11 detects sst2-expressing tumors with very high contrast (Fig 6). The HEK-hsst2 tumor had the highest tracer accumulation after 1, 4, and 24 hours p.i., compared with all non-tumor organs. Among normal organs, only the kidneys had a high radioactivity accumulation. At 24 hours p.i., the radioactivity from all of the other normal organs was completely cleared, while tumor still retained a significant amount of radioactivity for clear visualization. PET images (Fig 7) demonstrated that with increasing peptide mass, the background activity in the abdomen decreases but the kidneys still have a high amount of radioactivity. In addition, there was a significant reduction of activity in the tumor due to blocking effect at the peptide masses of 1000 pmol and above.

## Discussion

Neuroendocrine tumors are heterogeneous in the sense that they can arise in various organs of the body. They are also diverse with regard to their biology. But they share common features related to overexpression of different hormone receptors. In particular, somatostatin receptors (the most abundant is sst2) are present in high density and high incidence in differentiated NETs (Ki-67 < 22) [25]. Thus, they represent ideal targets for imaging and targeted radionuclide therapy. Indeed, several DOTA-conjugated octapeptides targeting somatostatin receptors, labeled with ^68^Ga (^68^Ga-DOTA-TOC, ^68^Ga-DOTA-NOC, and ^68^Ga-DOTA-TATE), are considered state-of-the-art for diagnosis, staging, therapy, and follow-up of patients with NETs [26].

Copper-64 has gained considerable recognition due to its good positron energy and a half-life of 12.7 hours. The longer half-life allows PET imaging at later time points than ^68^Ga (t_1/2_ = 68 minutes) with potentially higher tumor-to-normal organ contrast as well as central production and shipping to remote hospitals [10, 17, 18]. In addition, ^64^Cu-DOTA-TATE, for instance, was shown to outperform not only the SPECT tracer OctreoScan ([^111^In-DTPA]octreotide) in NET patients [21] but also one of the gold standard PET/CT agents: ^68^Ga-DOTA-TOC [20]. This comes as a surprise, as preclinical studies have indicated that DOTA is not a suitable chelator for Cu(II) radiopharmaceuticals because of some *in vivo* instability of this chelate, most likely due to reduction to Cu(I) and the instability of the Cu(I)-DOTA complex [27].

Furthermore, preclinical studies and preliminary clinical studies have shown that receptor antagonist-based tracers are superior to receptor agonists [5–8]. Among a series of antagonists, the octapeptide JR11 (p-Cl-Phe-cyclo(DCys-Aph(Hor)-DAph(Cbm)-Lys-Thr-Cys)DTyr-NH_2_) was selected for clinical translation. The DOTA- or NODAGA-conjugates of JR11, labeled with ^177^Lu and ^68^Ga, respectively, were studied in phase I and II clinical studies [28–30]. In the present study, we evaluated ^64^Cu-NODAGA-JR11 as an sst2-targeting PET ligand and compared it side by side with ^64^Cu-DOTA-TATE, the clinically proven radiopeptide.

Both peptides showed high sst2 affinity and the Bmax value for the antagonist was about 8.5-fold higher than the agonist. Most importantly, this phenomenon was also observed with human tumors when quantitative autoradiography in neuroendocrine tumor specimens and non-neuroendocrine tumors was studied with ^125^I-labeled agonists and antagonists, respectively (^125^I-DOTA-JR11 and ^125^I-Tyr^3^octreotide) [4].

The cellular retention *in vitro* indicates that a substantial amount of the radioactivity is retained in the cells: 80% of ^64^Cu-DOTA-TATE (as internalized) and 60% of ^64^Cu-NODAGA-JR11 (as surface-bound), which is very similar to what was found for other sst2 receptor radioantagonists [6].

*In vivo* studies indicate that ^64^Cu-NODAGA-JR11 shows more favorable pharmacokinetics than ^64^Cu-DOTA-TATE with longer retention of activity in the tumor and improved tumor-to-normal organ ratios over time. Low levels in the liver and spleen and rapid blood clearance indicates that ^64^Cu-NODAGA-JR11 is stable *in vivo* [27]. In addition, the good labeling yields at high specific activity are other indications that NOTA analogues are ideal bifunctional chelators for ^64^Cu. We previously reported on another ^64^Cu-based radiopeptide, ^64^Cu-NODAGA-LM3, which has high potential for clinical translation. It shows even higher tumor uptake, longer tumor retention, and better tumor-to-normal organ ratios than ^64^Cu-NODAGA-JR11 [19]. In addition, ^64^Cu-Sar-TATE (a macrohexaaza-bicyclic chelator that forms exceptionally stable Cu(II) complexes coupled to TATE, [Tyr^3^,Thr^8^]octreotide) performs very favorably compared to ^64^Cu-DOTA-TATE with regard to tumor-to-most normal organ ratios, particularly at 24 hours post-injection in A427-7 tumor-bearing mice [31]. All three ^64^Cu-labeled radiopeptides warrant further comparative evaluation and potential clinical studies.

Conversely, ^64^Cu-DOTA-TATE shows persistent blood and heart values over 24 hours. In addition, the high liver uptake and long liver retention compared with ^64^Cu-NODAGA-JR11 suggest tracer instability, as found by other researchers with different DOTA-conjugated tracers [32]. Despite these findings, ^64^Cu-DOTA-TATE is very successful in humans and appears to be stable with regard to ^64^Cu-ion release. This raises an interesting question of whether we are dealing with a species difference. One explanation could be a difference in reductant concentration in the blood of the two species (human and mouse). Among the most likely reducing agents in human/mouse plasma are low molecular weight biothiols such as Cys, hCys, Cysgly, and glutathione, which are present in high concentrations in both human and mouse plasma. The total thiol concentration in mouse plasma is about 320 μM, but in human plasma it is only 228 μM. Specifically, there is about an 80-fold higher glutathione concentration in mouse plasma [33,34].

## Conclusions

Independent of the mechanism, ^64^Cu-NODAGA-JR11 performs better than ^64^Cu-DOTA-TATE in this xenograft animal model and the bifunctional chelator NODAGA allows for the strong encapsulation of Cu^2+^ by the 3 nitrogens and 3 carboxy methyl oxygen atoms of the parent NOTA, while still providing a carboxy ethyl group for biomolecule coupling. The comparison of these two ^64^Cu-labeled radiopeptides allows us to state that ^64^Cu-NODAGA-JR11 warrants further development for translation into the clinic.

## Supporting information

S1 Fig **RadioHPLC chromatogram showing the radiochemical purity of the** ^**64**^**Cu-NODAGA-JR11 (A) and** ^**64**^**Cu-DOTA-TATE (B).** The values on the x-axis indicate retention time in minutes. Free ^64^Cu elutes earlier than 1 minute.
(PDF)

S2 Fig Internalization kinetics of ^64^Cu-DOTA-TATE in HEK293-hsst_2_ cells. (PDF)

## References

[1] de Jong M, Breeman WA, Bakker WH, Kooij PP, Bernard BF, Hofland LJ, et al Comparison of (111)In-labeled somatostatin analogues for tumor scintigraphy and radionuclide therapy. Cancer Res. 1998;58: 437–441. 9458086

[2] Waser B, Tamma ML, Cescato R, Maecke HR, Reubi JC. Highly efficient in vivo agonist-induced internalization of sst2 receptors in somatostatin target tissues. J Nucl Med. 2009;50(6): 936–941. doi: 10.2967/jnumed.108.061457 19443580 10.2967/jnumed.108.061457

[3] Deupi X. A stitch in time. Nat Chemistry. 2014;6: 7–8. 10.1038/nchem.1832 24345940

[4] Reubi JC, Waser B, Maecke HR, Rivier JE. Highly increased 125I-JR11 antagonist binding in vitro reveals novel indications for sst2 targeting in human cancers. J Nucl Med. 2017;58: 300–306. doi: 10.2967/jnumed.116.177733 27561878 10.2967/jnumed.116.177733

[5] Ginj M, Zhang H, Waser B, Cescato R, Wild D, Wang X, et al Radiolabeled somatostatin receptor antagonists are preferable to agonists for in vivo peptide receptor targeting of tumors. Proc Natl Acad Sci U S A. 2006;103: 16436–16441. doi: 10.1073/pnas.0607761103 17056720 10.1073/pnas.0607761103 PMC1618814

[6] Fani M, Del Pozzo L, Abiraj K, Mansi R, Tamma ML, Cescato R, et al PET of somatostatin receptor-positive tumors using ^64^Cu- and ^68^Ga-somatostatin antagonists: the chelate makes the difference. J Nucl Med. 2011;52: 1110–1118. doi: 10.2967/jnumed.111.087999 21680701 10.2967/jnumed.111.087999

[7] Wild D, Fani M, Behe M, Brink I, Rivier JE, Reubi JC, et al First clinical evidence that imaging with somatostatin receptor antagonists is feasible. J Nucl Med. 2011;52: 1412–1417. doi: 10.2967/jnumed.111.088922 21852357 10.2967/jnumed.111.088922

[8] Nicolas GP, Mansi R, McDougall L, Kaufmann J, Bouterfa H, Wild D, et al Biodistribution, pharmacokinetics, and dosimetry of (177)Lu-, (90)Y-, and (111)In-labeled somatostatin receptor antagonist OPS201 in comparison to the agonist (177)Lu-DOTATATE: the mass effect. J Nucl Med. 2017;58(9): 1435–1441. doi: 10.2967/jnumed.117.191684 28450554 10.2967/jnumed.117.191684

[9] Marciniak A, Brasun J. Somatostatin analogues labelled with cooper radioisotopes: current status. J Radioanal Nucl Chem. 2017;313: 279–289. doi: 10.1007/s10967-017-5323-x 28804185 10.1007/s10967-017-5323-x PMC5533839

[10] Shokeen M, Anderson CJ. Molecular imaging of cancer with Copper-64 radiopharmaceuticals and positron emission tomography (PET). Accounts Chem Res. 2009;42: 832–841. 10.1021/ar800255q PMC2748250 19530674

[11] McCarthy DW, Shefer RE, Klinkowstein RE, Bass LA, Margeneau WH, Cutler CS, et al Efficient production of high specific activity ^64^Cu using a biomedical cyclotron. Nucl Med Biol. 1997;24: 35–43. 9080473 10.1016/s0969-8051(96)00157-6

[12] Wadas TJ, Wong EH, Weisman GR, Anderson CJ. Copper chelation chemistry and its role in copper radiopharmaceuticals. Current Pharm Design. 2007;13: 3–16. 10.2174/138161207779313768 17266585

[13] Sprague JE, Peng Y, Fiamengo AL, Woodin KS, Southwick EA, Weisman GR, et al Synthesis, characterization and in vivo studies of Cu(II)-64-labeled cross-bridged tetraazamacrocycle-amide complexes as models of peptide conjugate imaging agents. J Med Chem. 2007;50: 2527–35. doi: 10.1021/jm070204r 17458949 10.1021/jm070204r

[14] Gotzmann C, Braun F and Bartholom MD. Synthesis, 64Cu-labeling and PET imaging of 1,4,7-triazacyclononane derived chelators with pendant azaheterocyclic arms. RSC Advances. 2016;6: 119–131.

[15] Ghosh SC, Pinkston KL, Robinson H, Harvey BR, Wilganowski N, Gore K, et al Comparison of DOTA and NODAGA as chelators for 64Cu-labeled immunoconjugates. Nucl Med Biol. 2015;43: 177–183. 10.1016/j.nucmedbio.2014.09.009 25457653

[16] Cooper MS, Ma MT, Sunassee K, Shaw KP, Williams JD, Paul RL et al Comparison of ^64^Cu-complexing bifunctional chelators for radioimmunoconjugation: labelling efficiency, specific activity and in vitro/ in vivo stability. Bioconj Chem. 2012;23: 1029–1039. 10.1021/bc300037w PMC4756438 22471317

[17] Hao G, Singh AN, Oz OK, Sun X Recent advances in copper radiopharmaceuticals. Current Radiopharm. 2011;4: 109–121. 10.2174/1874471011104020109 22191650

[18] Shokeen M, Waddas TJ. The development of copper radiopharmaceuticals for imaging and therapy. Med Chem. 2011;7: 413–429. 21711219 10.2174/157340611796799177 PMC8259694

[19] Fani M, Braun F, Waser B, Beetschen K, Cescato R, Erchegyi J, et al Unexpected sensitivity of sst2 antagonists to N-terminal radiometal modifications. J Nucl Med. 2012;53: 1481–1489 doi: 10.2967/jnumed.112.102764 22851637 10.2967/jnumed.112.102764

[20] Johnbeck CB, Knigge U, Loft A, Berthelsen AK, Mortensen J, Oturai P, et al Head-to-head comparison of (64)Cu-DOTATATE and (68)Ga-DOTATOC PET/CT: a prospective study of 59 patients with neuroendocrine tumors. J Nucl Med. 2017;58: 451–457. doi: 10.2967/jnumed.116.180430 27660147 10.2967/jnumed.116.180430

[21] Pfeifer A, Knigge U, Binderup T, Mortensen J, Oturai P, Loft A, et al 64Cu-DOTATATE PET for neuroendocrine tumors: a prospective head-to-head comparison with 111In-DTPA-octreotide in 112 patients. J Nucl Med. 2015;56: 847–54. doi: 10.2967/jnumed.115.156539 25952736 10.2967/jnumed.115.156539

[22] Cescato R, Erchegyi J, Waser B, Piccand V, Maecke HR, Rivier JE, et al Design and in vitro characterization of highly sst2-selective somatostatin antagonists suitable for radiotargeting. J Med Chem. 2008;51: 4030–4037. doi: 10.1021/jm701618q 18543899 10.1021/jm701618q PMC2789649

[23] Storch D, Béhé M, Walter MA, Chen J, Powell P, Mikolajczak R, et al Evaluation of [^99m^Tc/EDDA/HYNIC0]octreotide derivatives compared with [^111^In-DOTA^0^,Tyr^3^, Thr^8^]octreotide and [^111^In-DTPA0]octreotide: does tumor or pancreas uptake correlate with the rate of internalization? J Nucl Med. 2005;46: 1561–1569. 16157541

[24] Läppchen T, Tönnesmann R, Eersels J, Meyer PT, Maecke HR, Rylova SN. Radioiodinated exendin-4 is superior to the radiometal-labelled glucagon-like peptide-1 receptor probes overcoming their high kidney uptake. PLoS One. 2017;12: e0170435 doi: 10.1371/journal.pone.0170435 28103285 10.1371/journal.pone.0170435 PMC5245897

[25] Kunz PL. Carcinoid and neuroendocrine tumors: building on success. J Clin Oncol. 2015;33: 1855–63. doi: 10.1200/JCO.2014.60.2532 25918282 10.1200/JCO.2014.60.2532

[26] Smit Duijzentkunst DA, Kwekkeboom DJ, Bodei L. Somatostatin receptor 2-targeting compounds. J Nucl Med. 2017;58(Suppl 2): 54S–60S. doi: 10.2967/jnumed.117.191015 28864613 10.2967/jnumed.117.191015

[27] Woodlin SL, Heroux KJ, Boswell CA, Wong EH, Weisman GR, Niu W et al Kinetic inertness and electrochemical behavior of Copper (II) tetraazamacrocyclic complexes: possible implications for in vivo stability. Eur J Inorg Chem. 2005, 4829–4833.

[28] Wild D, Fani M, Fischer R, Del Pozzo L, Kaul F, Krebs S, et al Comparison of somatostatin receptor agonist and antagonist for peptide receptor radionuclide therapy: a pilot study. J Nucl Med. 2014;55: 1248–52. doi: 10.2967/jnumed.114.138834 24963127 10.2967/jnumed.114.138834

[29] Nicolas GP, Schreiter N, Kaul F, Uiters J, Bouterfa H, Kaufmann J, et al Comparison of (68)Ga-OPS202 ((68)Ga-NODAGA-JR11) and (68)Ga-DOTATOC ((68)Ga-Edotreotide) PET/CT in patients with gastroenteropancreatic neuroendocrine tumors: evaluation of sensitivity in a prospective phase II imaging study. J Nucl Med. 2017 11 30 pii: jnumed.117.199760. doi: 10.2967/jnumed.117.199760 29191855

[30] Nicolas GP, Beykan S, Bouterfa H, Kaufmann J, Bauman A, Lassmann M, et al Safety, biodistribution, and radiation dosimetry of (68)Ga-OPS202 ((68)Ga-NODAGA-JR11) in patients with gastroenteropancreatic neuroendocrine tumors: a prospective phase I imaging study. J Nucl Med. 2017 10 12 pii: jnumed.117.199737. doi: 10.2967/jnumed.117.199737 29025985 10.2967/jnumed.117.199737

[31] Paterson BM, Roselt P, Denoyer D, Cullinane C, Binns D, Noonan W, et al PET imaging of tumours with a 64Cu labeled macrobicyclic cage amine ligand tethered to Tyr3-octreotate. Dalton Trans. 2014;43: 1386–96. doi: 10.1039/c3dt52647j 24202174 10.1039/c3dt52647j

[32] Hu LY, Bauer N, Knight LM, Li Z, Liu S, Anderson CJ, et al Characterization and evaluation of (64)Cu-labeled A20FMDV2 conjugates for imaging the integrin αvβ 6. Mol Imaging Biol. 2014;16: 567–77. doi: 10.1007/s11307-013-0717-9 24448825 10.1007/s11307-013-0717-9 PMC4277820

[33] Isokawa M, Funatsu T, Tsunoda M. Fast and simultaneous analysis of biothiols by high performance liquid chromatography with fluorescence detection under hydrophilic interaction chromatography conditions. Analyst. 2013;138: 3802–3808. doi: 10.1039/c3an00527e 23702918 10.1039/c3an00527e

[34] Isakowa M, Shimosawa T, Funatsu T, Tsunoda M. Determination and characterization of total thiols in mouse serum samples using hydrophilic interaction liquid chromatography with fluorescence detection and mass spectrometry. J Chromatogr B. 2016;1019: 59–65. 10.1016/j.jchromb.2015.11.038 26691842

