# Supplementary material for: The somatostatin receptor 2 antagonist 64Cu-NODAGA-JR11 outperforms 64Cu-DOTA-TATE in a mouse xenograft model
Source: PLoS One. 2018 Apr 18;13(4):e0195802. doi: 10.1371/journal.pone.0195802 (PMC5906006; doi:10.1371/journal.pone.0195802)
Supplement: S1 Fig — RadioHPLC chromatogram showing the radiochemical purity of the 64Cu-NODAGA-JR11 (A) and 64Cu-DOTA-TATE (B). The values on the x-axis indicate retention time in minutes. Free 64Cu elutes earlier than 1 minute. (PDF) [file pone.0195802.s001.pdf]

Supporting Information

S1 Fig. RadioHPLC chromatogramm showing the radiochemical purity of the ^{64}Cu-NODAGA-JR11 (A) and ^{64}Cu-DOTA-TATE (B). The values on the x-axis indicate the retention time in minutes. Free ^{64}Cu elutes earlier than 1 minute.

A

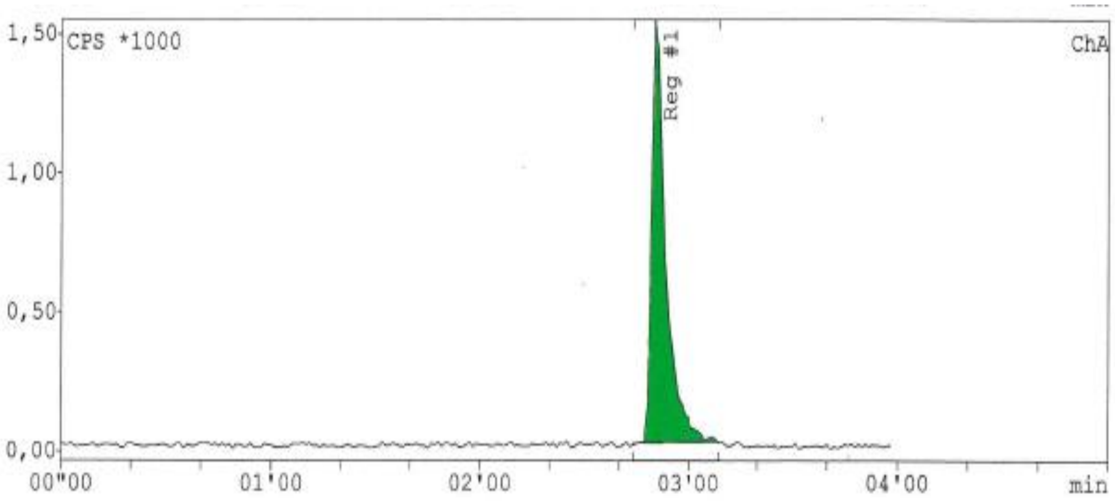

B

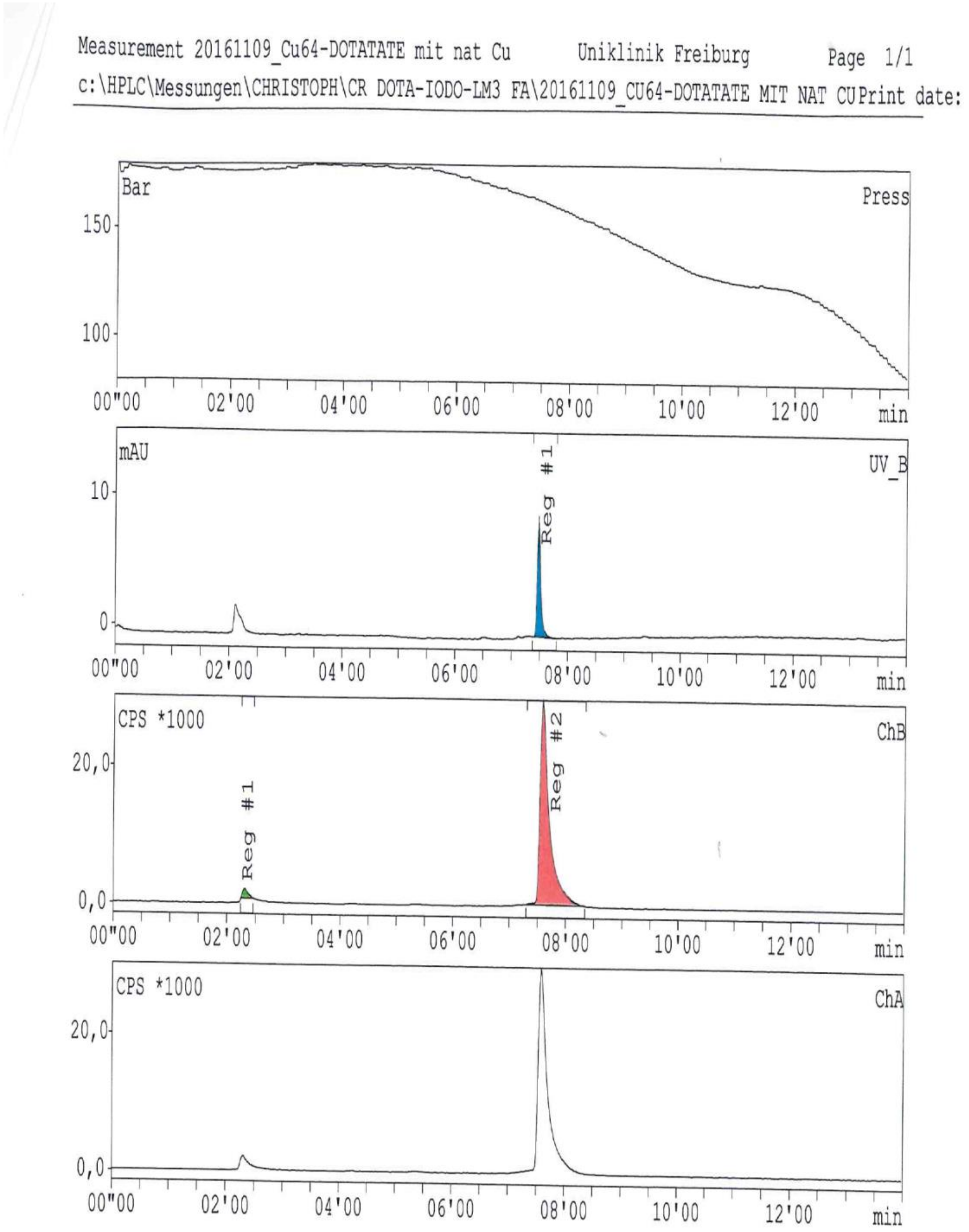
Measurement 20161109_Cu64-DOTATATE mit nat Cu
Uniklinik Freiburg
Page 1/1
c:\HPLC\Messungen\CHRISTOPH\CR DOTA-IODO-LM3 FA\20161109_CU64-DOTATATE MIT NAT CU
Print date:
Bar
Press
150
100
mAU
UV_B
10
0
Reg #1
CPS *1000
ChB
20,0
0,0
Reg #1
Reg #2
CPS *1000
ChA
20,0
0,0
00"00
02'00
04'00
06'00
08'00
10'00
12'00
min
