# Supplementary material for: The somatostatin receptor 2 antagonist 64Cu-NODAGA-JR11 outperforms 64Cu-DOTA-TATE in a mouse xenograft model
Source: PLoS One. 2018 Apr 18;13(4):e0195802. doi: 10.1371/journal.pone.0195802 (PMC5906006; doi:10.1371/journal.pone.0195802)

Supporting Information

S2 Fig. Internalization kinetics of ^{64}Cu-DOTA-TATE in HEK293-hsst$_2$ cells.

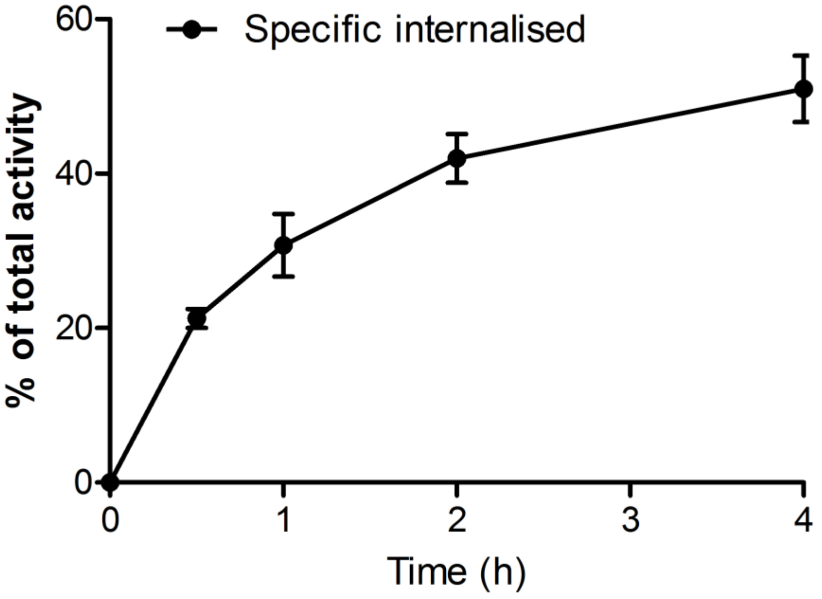

Supplement: S2 Fig — (PDF) [file pone.0195802.s002.pdf]
